# Do carnivorous plants exploit prey perceptual bias? A case study in *Drosera finlaysonii* with yellow-green traps

**DOI:** 10.1098/rsos.261740

**Published:** 2026-10-28

**Authors:** Kazuki Tagawa, Aoi Watanabe, Shu-Miaw Chaw, Mitsuyasu Hasebe, Mikio Watanabe

**Affiliations:** ^1^ Department of Biology, Naruto University of Education, Naruto, Japan; ^2^ Department of Economics, Fukui Prefectural University, Yoshida, Japan; ^3^ Biodiversity Research Center, Academia Sinica, Taipei City, Taiwan; ^4^ Basic Biology Program, Graduate University for Advanced Studies (SOKENDAI), Okazaki, Japan; ^5^ National Institute for Basic Biology, Okazaki, Japan; ^6^ Department of Biology, Aichi University of Education, Kariya, Japan

**Keywords:** carnivorous plants, *Drosera*, exploitation of perceptual bias, plant–animal communication

## Abstract

Perceptual biases—disproportionate sensory responses to specific stimuli—are widespread in animals and can be exploited by predators to attract prey. However, this mechanism has not been rigorously tested in carnivorous plants. We investigated whether the yellow-green traps of *Drosera finlaysonii* (Droseraceae) exploit hemipterans’ preference for yellow to yellow-green stimuli that strongly activate green relative to blue photoreceptors. Using multispectral imaging, we quantified trap brightness and modelled the green-to-blue photoreceptor activation ratio (G/B ratio) of hemipteran prey. We characterized the taxonomic composition of prey captured by *D. finlaysonii* and deployed adhesive models differing in brightness and G/B ratio. We then compared arthropod assemblages between plants and models, and taxon-specific capture numbers among models. *D. finlaysonii* traps were brighter and had higher G/B ratios than sympatric graminoid leaves. Hemiptera were a major prey component, although their relative abundance varied between years. Yellow models, with the highest G/B ratio, captured more hemipterans than other colour models. However, arthropod assemblages captured by *D. finlaysonii* differed from those captured by yellow models, with proportionally more dipterans and fewer hemipterans. Thus, a high G/B ratio may contribute to hemipteran attraction and capture but cannot alone explain the overall prey assemblage in *D. finlaysonii*.

## 1. Introduction

Organisms do not perceive their environment as an objective replica of physical reality. Instead, they experience a species- and individual-specific perceptual world shaped by the constraints of their sensory receptors and cognitive processing systems. Such sensory and cognitive characteristics can generate perceptual biases, defined as disproportionate responses to particular classes of stimuli [1]. When these biases enhance fitness by improving foraging efficiency or reproductive success, the underlying perceptual traits are expected to be maintained by natural selection.

In antagonistic interactions between species, senders may exploit pre-existing perceptual biases of receivers to increase the effectiveness of their signals—a strategy known as the exploitation of perceptual biases (EPB). This phenomenon has been documented across a wide range of interactions. For example, some sit-and-wait predators possess traits that closely match the perceptual biases prey have evolved for resource detection, thereby creating stimuli that are difficult for prey to ignore [2,3]. Unlike mimicry, EPB does not require the existence of a specific model. Consequently, it is subject to fewer evolutionary constraints and is thought to represent a more universal and evolutionarily accessible form of deception than mimicry [1].

Carnivorous plants may also employ prey-attraction mechanisms that function through EPB. They are adapted to nutrient-poor environments and enhance their fitness by capturing prey and allocating the acquired nutrients to increase photosynthetic rates and seed production [4,5]. Accordingly, traits that attract prey through EPB and increase capture success are expected to be evolutionarily favoured. Possible examples of such traits include the dark central regions, radiating stripes and peripheral dots commonly found on the trap surfaces of pitcher plants. Biesmeijer *et al.* [6] proposed that these traits evolved under selection to enhance prey attraction by exploiting perceptual biases in bees, based on both the frequent occurrence of these patterns in three phylogenetically independent lineages of pitcher plants (i.e. *Nepenthes*, *Sarracenia* and *Cephalotus*) and the attraction of bees to similar patterns. They suggested that responses to dark centres and radiating stripes aid nest localization, whereas responses to dark dots promote mate-seeking and aggregation, implying that these perceptual biases evolved in bees before being exploited by pitcher plants. More broadly, many carnivorous plants—including species with pitfall and adhesive traps—exhibit pronounced visual contrast patterns based on ultraviolet (UV) reflectance and absorption. For instance, in several *Nepenthes* species, the pitcher body reflects UV, whereas the nectar-secreting pitcher rim (peristome) absorbs UV [7,8]. Such patterns may exploit the perceptual bias of insects to detect UV contrast and orient toward particular contrast configurations [9]. Consistent with this possibility, Moran [10] demonstrated that *N. rafflesiana*, which exhibits a strong UV contrast between the pitcher body and rim, attracts and captures a broader range of insect taxa including anthophiles than the sympatric *N. gracilis*, which shows weaker contrast. Although these contrast patterns closely resemble those commonly found in flowers [11], no specific model used as a reference by receivers has been identified, and there is no evidence supporting floral mimicry by carnivorous plants [12,13]. Rather, these examples suggest that flowers and carnivorous plant traps may have convergently evolved to exploit similar perceptual biases in insects [6,14].

Nevertheless, the previous empirical studies have not yet provided rigorous tests of EPB in carnivorous plants. Biesmeijer *et al.* [6] examined bee responses, but whether the visual patterns described above also attract ants and flies, the predominant prey of many pitcher plant species [4], remains unclear. In addition, because the two *Nepenthes* species compared by Moran [10] may differ in traits other than UV contrast, the observed difference in prey capture cannot be attributed unequivocally to the focal visual traits.

We propose that a rigorous test of EPB in a predator–prey interaction should establish the following four criteria (table 1). First, the target prey must possess a pre-existing perceptual bias towards a particular stimulus that arose independently of its interaction with the predator. Second, the predator must possess a trait that produces a stimulus matching the prey's perceptual bias. Third, the predator must be shown to capture the target prey under natural field conditions. Fourth, experimental manipulation of the focal trait or stimulus must alter the capture rate of the target prey in the predicted direction. To apply this four-criterion framework to EPB in carnivorous plants, we focused on the interaction between *Drosera finlaysonii* (Droseraceae), a sundew that produces slender aerial adhesive trap leaves, and herbivorous hemipteran insects. In its habitat, the trap leaves of *D. finlaysonii* are conspicuously bright yellow-green and contrast strongly with the green foliage of the surrounding vegetation (figure 1*a*). During preliminary field observations, we also found entire trap leaves of *D. finlaysonii* covered with hemipteran insects, including leafhoppers (Hemiptera: Auchenorrhyncha; figure 1*b*) and whiteflies (Hemiptera: Sternorrhyncha; figure 1*c*). Based on these observations, we hypothesized that the yellow-green traps of *D. finlaysonii* exploit a perceptual bias in hemipterans and thereby enhance the attraction and capture of hemipterans.

**Table 1.** Four criteria proposed for testing the exploitation of perceptual bias (EPB) and their application in this study.

criteria for EPB	the proposed framework	application in this study (*D. finlaysonii* and Hemiptera)	addressed in
criterion 1	target prey has a pre-existing perceptual bias towards a particular stimulus	hemipteran herbivores show a pre-existing preference for yellow stimuli with a high G/B ratio	e.g. [15,16]
criterion 2	predator possesses a trait that produces a stimulus matching the bias of prey	the traps of *D. finlaysonii* display a bright yellow-green colour with a high G/B ratio	study (*a*)
criterion 3	predator captures the target prey under natural field conditions	*D. finlaysonii* captures hemipterans under natural field conditions	study (*b*)
criterion 4	experimental manipulation of the focal trait or stimulus alters capture rates in the predicted direction	artificial traps differing in G/B ratio show predicted differences in Hemiptera capture rates	study (*c*)

**Figure 1.**
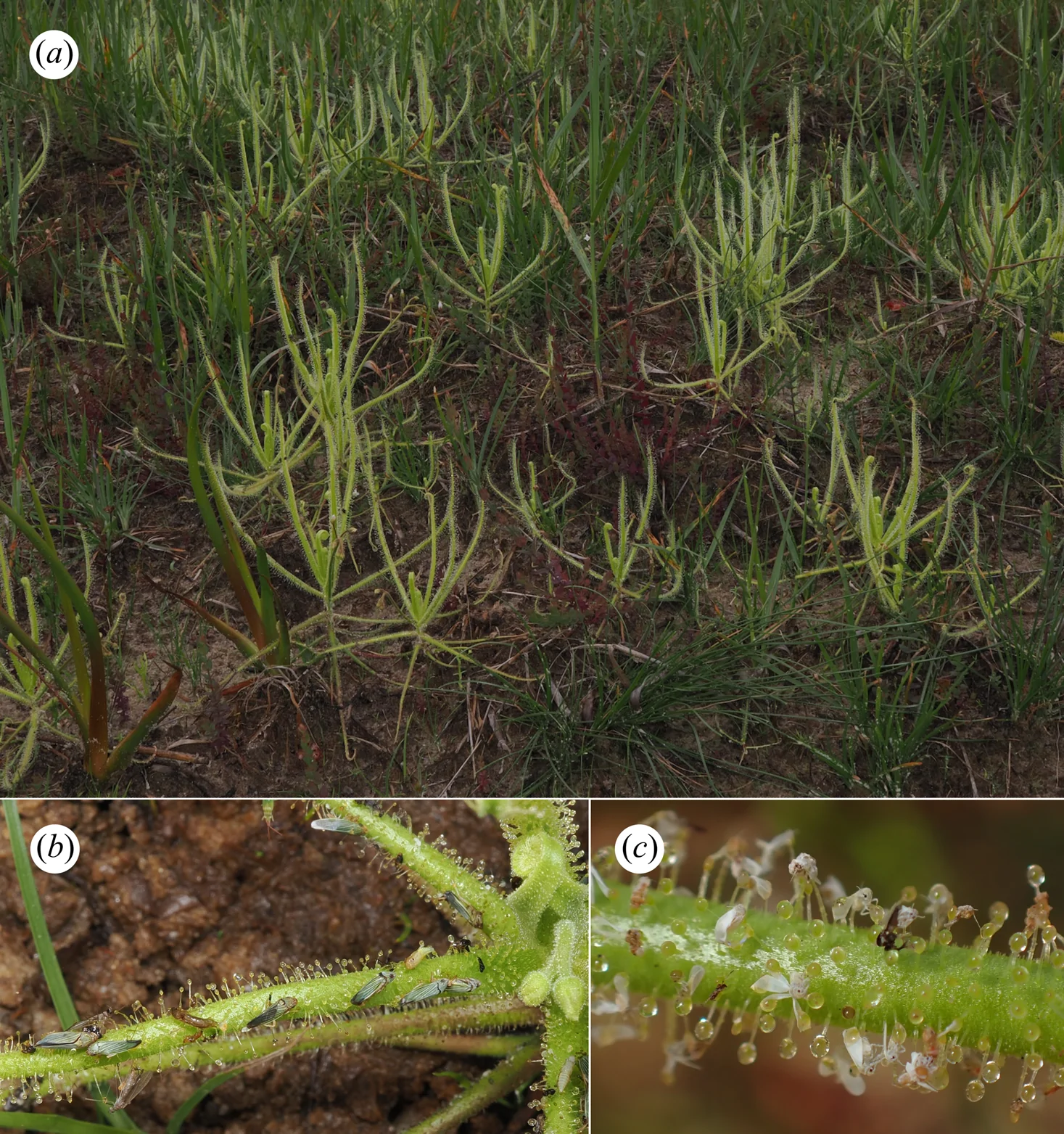
*Drosera finlaysonii* and trapped prey. (*a*) *D. finlaysonii* possesses bright yellow-green trap leaves that are visually conspicuous compared with the leaves of sympatric graminoid species. (*b*) Leafhoppers (Hemiptera: Auchenorrhyncha) trapped by *D. finlaysonii*. (*c*) Whiteflies (Hemiptera: Sternorrhyncha) trapped by *D. finlaysonii*.

With respect to criterion 1, the preference of herbivorous insects for the colour yellow is a well-established pre-existing perceptual bias. Most insects possess three types of photoreceptors sensitive to UV, blue (B) and green (G) wavelengths [17]. The green receptor typically operates antagonistically to the blue and UV receptors through a colour-opponent mechanism. Herbivorous insects primarily rely on the green receptor to locate host plant foliage; however, objects with high reflectance in the yellow range strongly stimulate the green receptor while suppressing excitation of the blue and UV receptors. As a result, yellow stimuli are perceived as supernormal foliage-type stimuli that are even more attractive than green leaves [15,18,19]. Empirically, many hemipteran herbivores—including aphids, whiteflies and psyllids—exhibit a consistent preference for yellow over green [16,20–22]. Moreover, attraction effectiveness has been shown to correlate positively with the activation ratio of green to blue photoreceptors of aphids (the G/B ratio [23]). This perceptual bias is thought to have been maintained by natural selection because it enhances the fitness of the herbivores. Young leaves in which chlorophyll accumulation is incomplete and senescing leaves in which chlorophyll has degraded typically have elevated concentrations of soluble nitrogen (e.g. amino acids) in their phloem. Because phloem-feeding insects rely directly on phloem sap as their primary nutritional source, such leaves represent particularly high-quality resources for them [18,24] (but see [25]).

In the present study, we tested the remaining three criteria to evaluate EPB in *D. finlaysonii* (table 1). Specifically, we addressed the following questions:

— Criterion 2: Do the traps of *D. finlaysonii* have higher G/B ratios than the surrounding vegetation?
— Criterion 3: Does *D. finlaysonii* capture hemipterans under natural field conditions?
— Criterion 4: Does hemipteran capture vary predictably among artificial traps with different G/B ratios?

Through these analyses, we evaluate whether *D. finlaysonii* enhances prey capture efficiency via EPB mediated by variation in the G/B ratio, and discuss the implications of this mechanism for the evolutionary ecology of carnivorous plants.

## 2. Material and methods

### 2.1. Study species

*Drosera finlaysonii* Wall. ex Arn., syn. *D. finlaysoniana* Wall. ex Stein (Droseraceae) is an annual carnivorous plant belonging to *Drosera* section *Arachnopus* (i.e. *D. indica* complex). This species grows up to approximately 30 cm in height and is characterized by slender, aerial adhesive trap leaves. Based on our preliminary observations, *D. finlaysonii* appears to differ from other Asian species of *Drosera* section *Arachnopus* by forming relatively short tentacles at high density. In addition, yellowish granular structures are present on the tentacle stalks (supplementary material, figure S1a,b), causing the trap leaves to appear yellow to whitish when viewed laterally. The species is distributed across Australia, China, Taiwan and Vietnam [26]. While it is widespread from northern to central Australia, its Asian distribution is restricted to coastal regions along the South China Sea.

### 2.2. Study site

Fieldwork was conducted in a coastal sandy wetland on Kinmen Island, Taiwan. Because *D. finlaysonii* is listed as an endangered species, precise geographic coordinates are not provided to protect the natural population. The habitat exhibits a pronounced environmental gradient ranging from permanent ponds to dry grassland. Within an area of approximately 1200 m², more than 1000 individuals of *D. finlaysonii* grow naturally. Seeds typically germinate in March, with flowering commencing around May and continuing until approximately October.

### 2.3. Quantification of visual traits

Because many insects captured by *D. finlaysonii* are capable of UV vision, we quantified visual traits including the UV range. To achieve this, we employed full-spectrum digital photography and multispectral imaging. Traditionally, reflectance spectra have been measured using spectrophotometers; however, this approach requires point-by-point contact measurements and is poorly suited for evaluating how entire trap leaves appear to insects at a broader spatial scale. Moreover, spectrophotometric measurements are sensitive to probe angle and the distance between the probe and the sample [3,27], and typically require controlled indoor conditions. These constraints make spectrophotometry impractical for studies of endangered species for which destructive sampling should be avoided. By contrast, multispectral digital photography overcomes these limitations by enabling the analysis of large-scale visual patterns across entire plants using non-invasive field measurements [27,28].

Images were captured using a full-spectrum-modified SONY α6000 camera equipped with a SIGMA 50 mm lens [29,30], allowing the recording of both UV and visible wavelength ranges. Photography was conducted under clear-sky conditions between 07.00 and 08.00 in May 2025 at the study site. For each scene, two consecutive images were taken using a UV/IR-cut filter and a UV-pass + IR-cut filter to obtain images in the visible (400–700 nm) and UV (300–400 nm) ranges, respectively. Camera settings were fixed at f/5.0 and ISO 200, while shutter speed was adjusted *in situ* to ensure appropriate exposure. Each image frame included at least one individual of *D. finlaysonii*, leaves of sympatric graminoid species (i.e. Poaceae and Cyperaceae), and background soil. An 18% grey standard and a scale bar were included in all images for calibration purposes, and all images were saved in RAW format.

Image processing was conducted using the MICA (Multispectral Image Calibration and Analysis) plugin [27] for ImageJ [31]. MICA performs radiometric calibration based on the grey standard and generates multispectral images consisting of three visible channels (vR, vG and vB) and two UV channels (uB and uR). This procedure enables quantitative comparisons of colour and pattern both within and among images. For quantitative analyses, two regions of interest (ROIs) were selected for each image: (i) leaves of *D. finlaysonii* and (ii) those of surrounding graminoid species.

To test the hypothesis that *D. finlaysonii* trap leaves are brighter than surrounding vegetation, we first quantified brightness as a physical, receptor-independent property. Brightness was calculated as the mean reflectance across four channels (vR, vG, vB and uR), representing achromatic luminance [32,33]. Higher values indicate greater brightness. We compared both brightness and UV reflectance (uR), which is known to influence insect visual biases, between *D. finlaysonii* and graminoid leaves.

Next, to test the hypothesis that the visual traits of *D. finlaysonii* exploit hemipteran perceptual biases, we modelled trap-leaf appearance using the visual system of the green peach aphid, *Myzus persicae*, for which visual physiology and colour-choice behaviour are comparatively well characterized. Multispectral images were converted into predicted photoreceptor responses (cone catch values) using published spectral sensitivity data for *M. persicae* [16] and the camera sensitivity functions implemented in the MICA toolbox. Based on previous studies showing a positive correlation between attraction and the activation ratio of green to blue photoreceptors of aphids (G/B ratio) [23], we compared the G/B ratios of *D. finlaysonii* and graminoid leaves.

Whiteflies, aphids and leafhoppers were the major hemipterans trapped by *D. finlaysonii*. Representative species of all three groups possess visual channels sensitive to UV, blue and green wavelengths and respond to yellow or yellow-green vegetation-associated stimuli. Whiteflies also show evidence of blue–green opponency similar to that proposed for aphids [21,34]. By contrast, this mechanism has not been demonstrated in leafhoppers, for which overall reflectance intensity may also be important in host-foliage selection [35]. We therefore treated the aphid model as a qualitative proxy rather than a taxon-specific model for all hemipterans and considered overall reflectance intensity (i.e. brightness) separately when interpreting leafhopper attraction.

To test whether brightness, UV reflectance, and the G/B ratio differed between *D. finlaysonii* and graminoid leaves, we used generalized linear models (GLMs). In each model, the response variable was the respective visual trait, plant type was included as the explanatory variable, and a Gaussian error distribution and an identity link function were used.

Because the multispectral photography was conducted only under clear-sky conditions between 07.00 and 08.00, we could not quantify possible effects of cloud cover or solar elevation on the estimated visual traits [36]. However, *D. finlaysonii* and graminoid leaves were photographed simultaneously within the same images and normalized against the same grey reflectance standard, allowing standardized comparisons between the two plant types [27]. We also note that multispectral photography was conducted in May, whereas prey were sampled in September. Although we observed no obvious change in the visible appearance of *D. finlaysonii* between these periods, the seasonal stability of its multispectral properties, including UV reflectance and the aphid-model G/B ratio, remains to be tested quantitatively.

### 2.4. Survey of prey fauna

Prey captured by *D. finlaysonii* were recorded over three consecutive days in September 2024 and September 2025. In total, 20 plants were surveyed in September 2024 and 27 plants in September 2025. All prey items were documented exclusively through field photography. Traditionally, prey fauna of carnivorous plants have been assessed by collecting prey items or entire trap leaves and examining them in the laboratory (e.g. [37,38]). However, such approaches inevitably cause damage to the plants and are therefore unsuitable for studies focusing on species of conservation concern. Recent studies have demonstrated that non-invasive identification based on high-resolution digital images provides sufficient taxonomic resolution, at least at the order level [39,40]. To minimize damage to *D. finlaysonii*, we adopted this photographic approach. During surveys, flowering individuals were randomly selected while carefully avoiding trampling the surrounding vegetation. Each plant was photographed using an Olympus OM-1 camera equipped with an Olympus M.Zuiko Digital 30 mm macro lens and a twin flash system (Olympus, STF-8). All active trap leaves on each plant were photographed, and additional close-up images were taken when necessary to capture diagnostic morphological characters of the prey. Aperture was set between f/16 and f/22 to ensure sufficient depth of field for taxonomic identification.

Captured prey items were identified primarily to the family level from photographs. When identification to family was impossible, prey species were classified as morphospecies based on observable morphological features, or identified to the order level when only higher-level characters were preserved. A total of 1375 prey items were recorded in September 2024 and 893 in September 2025. Among these, 1365 items in 2024 and 773 items in 2025 retained sufficient morphological characters for at least order-level identification, as digestion on the trap leaves had not progressed substantially. These datasets were used for all subsequent analyses.

To test whether the number of captured individuals of the two major prey groups, Diptera and Hemiptera, differed between years, we used generalized linear models (GLMs). In each model, the response variable was the number of Diptera or Hemiptera individuals captured per plant, and year was included as the explanatory variable. Because models with a Poisson error distribution and a log link function showed overdispersion, statistical significance was assessed using models with a negative binomial error distribution and a log link.

### 2.5. Testing visual attraction using trap models

To examine whether *D. finlaysonii* attracts prey through specific visual traits, we deployed plant models bearing adhesive traps in the habitat and analysed the composition of captured prey. Trap models were constructed by printing specific colours onto matte cardboard sheets (10 × 1.5 cm) using an inkjet printer (Canon, TR703A), which were then covered with a transparent adhesive sheet designed for insect trapping (GeeRic, insect trap). Four trap colours were used: green, yellow-green, yellow and white. Green, yellow-green and yellow were selected to test whether differences in the G/B ratio influence insect attraction. Multispectral imaging using a full-spectrum camera confirmed that these trap models differed clearly in G/B ratio (green: 1.47; yellow-green: 2.19; yellow: 3.24; supplementary material, figure S2). Among these, the yellow trap models closely matched the G/B ratio of *D. finlaysonii* trap leaves (2.90 ± 0.49, mean ± s.d.), whereas the yellow-green trap models were similar to the sympatric dominant graminoid leaves (2.49 ± 0.50; supplementary material, figure S2). Therefore, if the G/B ratio influences prey composition in *D. finlaysonii*, we expected the yellow trap models to capture prey assemblages more similar to those observed on *D. finlaysonii* than the green or yellow-green traps. White traps were included to test the effect of high brightness. Although white traps differed markedly from *D. finlaysonii* in G/B ratio, they exhibited the highest brightness among all models (supplementary material, figure S2). If brightness alone plays a dominant role in prey attraction, white traps were expected to capture prey assemblages similar to those of *D. finlaysonii*. We selected trap models that were wider than trap leaves of *D. finlaysonii*. This choice was based on the assumption that the number of insects captured by the models would correlate with model surface area; using wider models was therefore expected to maximize the number of taxa and individuals captured within a single day, which was necessary for robust community analyses. However, it should be noted that, because of this choice, the trap models did not fully replicate the trap leaf morphology of *D. finlaysonii*. Trap models were attached to green plant models made of polylactic acid (PLA; height: 14 cm) that were produced using a 3D printer (Bambu Lab, A1; supplementary material, figure S3a). Each plant model held four trap models of the same colour, positioned at an angle of 30°, approximating the orientation of *D. finlaysonii* trap leaves.

In September 2025, seven blocks were established in the field, each consisting of one set of plant models representing the four trap colours (green, yellow-green, yellow and white; supplementary material, figure S3a,b). Blocks were spaced at least 2 m apart, and the positions of plant models within each block were randomized. Plant models were deployed at 08.00 and retrieved after 8 hours. Trap models were collected individually, sealed in plastic bags, and transported to the laboratory. The experiment was conducted over two consecutive days, yielding 14 replicate samples per colour. Trap models were then photographed in the laboratory, and captured insects were identified to the family level where possible, or classified as morphospecies when finer identification was not feasible. A total of 792 prey items were recorded and used for subsequent analyses, excluding ants. Many ants were captured on the trap models, often while attempting to remove captured insects, whereas ant kleptoparasitism on trap leaves of *D. finlaysonii* was observed only once during the study period. Preliminary behavioural observations suggest that mucilage secreted by tentacles on main stems of *D. finlaysonii* suppresses ant access to trap leaves, while ants can readily approach trap models on plant models lacking sticky stems. Accordingly, ant capture on models likely reflects structural differences rather than visual cues, and ants were excluded from our analyses of visual attraction. Although ants could potentially have deterred other insects from landing on or moving across the models, the effects of ants on the taxonomic composition of captured prey were likely limited. Most ants became immobilized near the margins of the trap models and rarely reached the central adhesive surfaces, where most flying prey were captured. Moreover, because ants were captured on trap models of all colours, any interference caused by their presence was unlikely to have systematically biased comparisons of prey assemblages among trap model colours.

To compare taxonomic compositions between years and among *D. finlaysonii* and the plant-model treatments, we constructed a dissimilarity matrix based on the Morisita index [41]. Using this matrix, we performed a non-metric multidimensional scaling (NMDS) analysis with two dimensions (*k* = 2) to visualize differences in prey assemblages among samples. To identify taxonomic gradients contributing to variation in prey composition, we calculated vectors for each taxon using the envfit function with 999 permutations and overlaid them onto the NMDS ordination. Prey taxa were generally classified at the order level. However, following previous studies [39], two major taxonomic groups were subdivided to achieve finer resolution: Diptera were divided into Brachycera and Nematocera, and Hemiptera were divided into Auchenorrhyncha, Heteroptera and Sternorrhyncha. The Morisita index was selected because it is relatively insensitive to differences in total abundance among samples, making it suitable for datasets in which sample sizes vary substantially [42], as in comparisons between *D. finlaysonii* and plant models. The statistical significance of clustering patterns observed in the NMDS ordination was tested using permutational multivariate analysis of variance (PERMANOVA) with 9999 permutations. Pairwise comparisons were subsequently conducted with Holm-adjusted *p*-values to account for multiple testing. Because PERMANOVA is known to be sensitive not only to differences in group centroids (location) but also to differences in dispersion among groups, we first assessed homogeneity of multivariate dispersion using permutational analysis of multivariate dispersion from group centroids (PERMDISP). As dispersion differed significantly among groups, we further evaluated differences in group location while explicitly considering dispersion effects by calculating Euclidean distances between group centroids in the NMDS space. To quantify the distinctiveness of prey assemblages for each group, we calculated the mean Euclidean distance between each group and all other groups (e.g. for the green model, the mean of the green–white, green–yellow and green–yellow-green distances). In addition, to test whether total prey abundance and the capture of major prey groups (Diptera, Hemiptera and Thysanoptera) differed among trap models, we constructed generalized linear mixed models (GLMMs). The response variable was the number of individuals captured per plant model; trap colour was included as a fixed effect, and block was treated as a random effect. Models were fitted with a Poisson error distribution and a log link function. No overdispersion was detected in these models. Multiple comparisons among trap colours were adjusted using the Holm method.

All statistical analyses in this study were conducted in R [43] and RStudio [44] using the packages car [45], dplyr [46], ggplot2 [47], ggthemes [48], lme4 [49], multcomp [50], performance [51], RColorBrewer [52], smplot [53], tibble [54], tidyr [55] and vegan [56].

## 3. Results

### 3.1. Visual traits

Multispectral imaging revealed that the trap leaves of *D. finlaysonii* were brighter than the surrounding graminoid leaves in both the visible and UV ranges (figure 2*a,b*). Consistent with these images, receptor-independent image analyses showed that the brightness of *D. finlaysonii* trap leaves was significantly higher than that of graminoid leaves across the full spectral range, including both visible and UV wavelengths (figure 3*a*), as well as in the UV wavelength alone (figure 3*b*). When prey perception was modelled using an aphid visual system, the trap leaves of *D. finlaysonii* were clearly distinguished from the graminoid leaves (figure 2*c*), and the green-to-blue photoreceptor activation ratio (G/B ratio) calculated from cone catch values was significantly higher for *D. finlaysonii* trap leaves than for graminoid leaves (figure 3*c*). This result indicates that the trap leaves of *D. finlaysonii* possess visual properties expected to elicit strong attraction responses in hemipteran insects.

**Figure 2.**
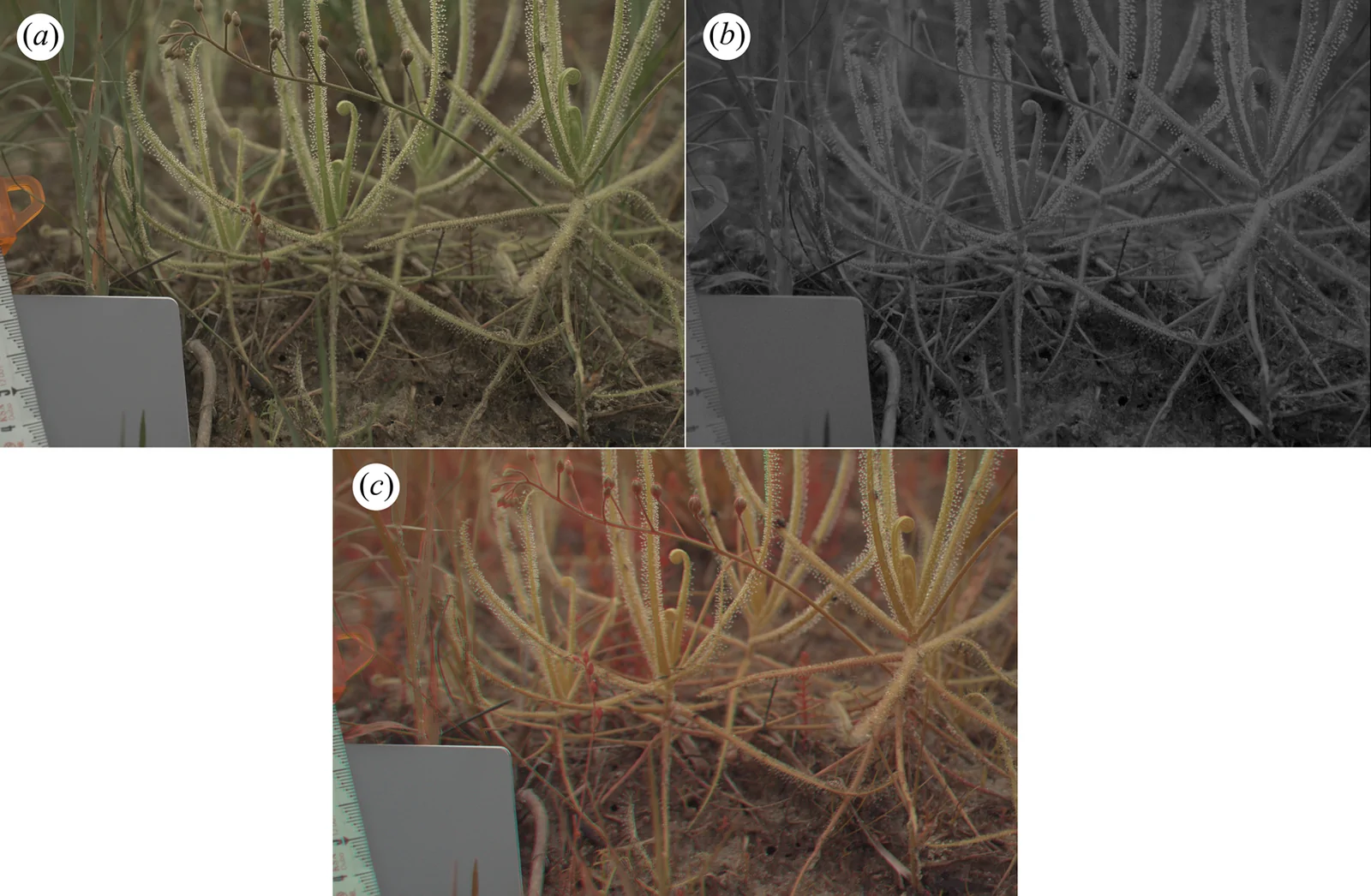
Receiver-specific appearances of *Drosera finlaysonii* trap leaves*.* (*a*) Image in the human visible spectrum (vR, vG and vB). (*b*) Image in the UV spectrum (uR and uB). (*c*) False-colour image representing the visible spectrum of an aphid (*Myzus persicae*). Outputs of the UV, G and B photoreceptors derived from square-root-transformed linear cone-catch images using the image calibration and analysis toolbox (MICA toolbox) are represented by red, blue and green, respectively.

**Figure 3.**
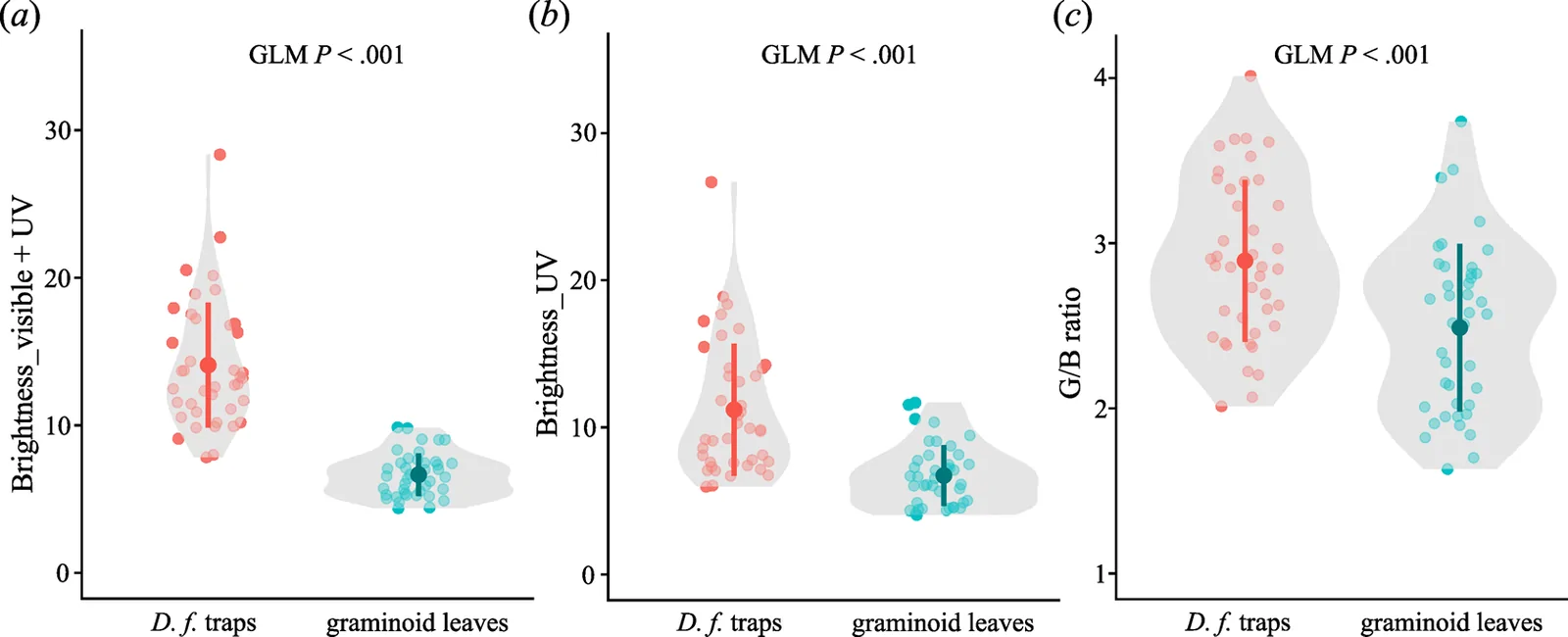
Visual characteristics of the trap leaves of *Drosera finlaysonii* and leaves of sympatric graminoid species. (*a*) Comparison of brightness, calculated as the mean reflectance across four channels (vR, vG, vB and uR). (*b*) Comparison of brightness in the UV range, based on uR reflectance. (*c*) Comparison of the ratio of green to blue photoreceptor cone catches (G/B ratio) under an aphid (*Myzus persicae*) visual system.

### 3.2. Prey fauna

In both 2024 and 2025, the majority of prey captured by *D. finlaysonii* consisted of Diptera and Hemiptera (figure 4*a*; supplementary material, table S1). In 2024, Hemiptera accounted for the highest proportion of captured prey items (68.1%), followed by Diptera (30.7%). Within Hemiptera, whiteflies (Aleyrodidae; Sternorrhyncha) were the dominant group, comprising 64.8% of all captured individuals, and at least one individual was recorded on every sampled plant. Within Diptera, biting midges (Ceratopogonidae; Nematocera) were the most abundant, accounting for 15.3% of all prey items. By contrast, in 2025 Diptera represented the largest proportion of captured prey items (64.7%), followed by Hemiptera (30.7%). As in 2024, Ceratopogonidae dominated the Diptera assemblage (55.4%), and Aleyrodidae were the most abundant hemipteran group (28.6%). Reflecting the reversal in the relative dominance of Hemiptera and Diptera between years, the overall taxonomic composition of the prey differed significantly between 2024 and 2025 (figure 4*b*; table 2). When the number of captured individuals per plant was compared between years, Diptera showed no statistically significant difference (GLM, χ² = 0.49, *p* = 0.48). By contrast, significantly more hemipteran individuals were captured in 2024 than in 2025 (GLM, χ² = 37.60, *p* < 0.001).

**Figure 4.**
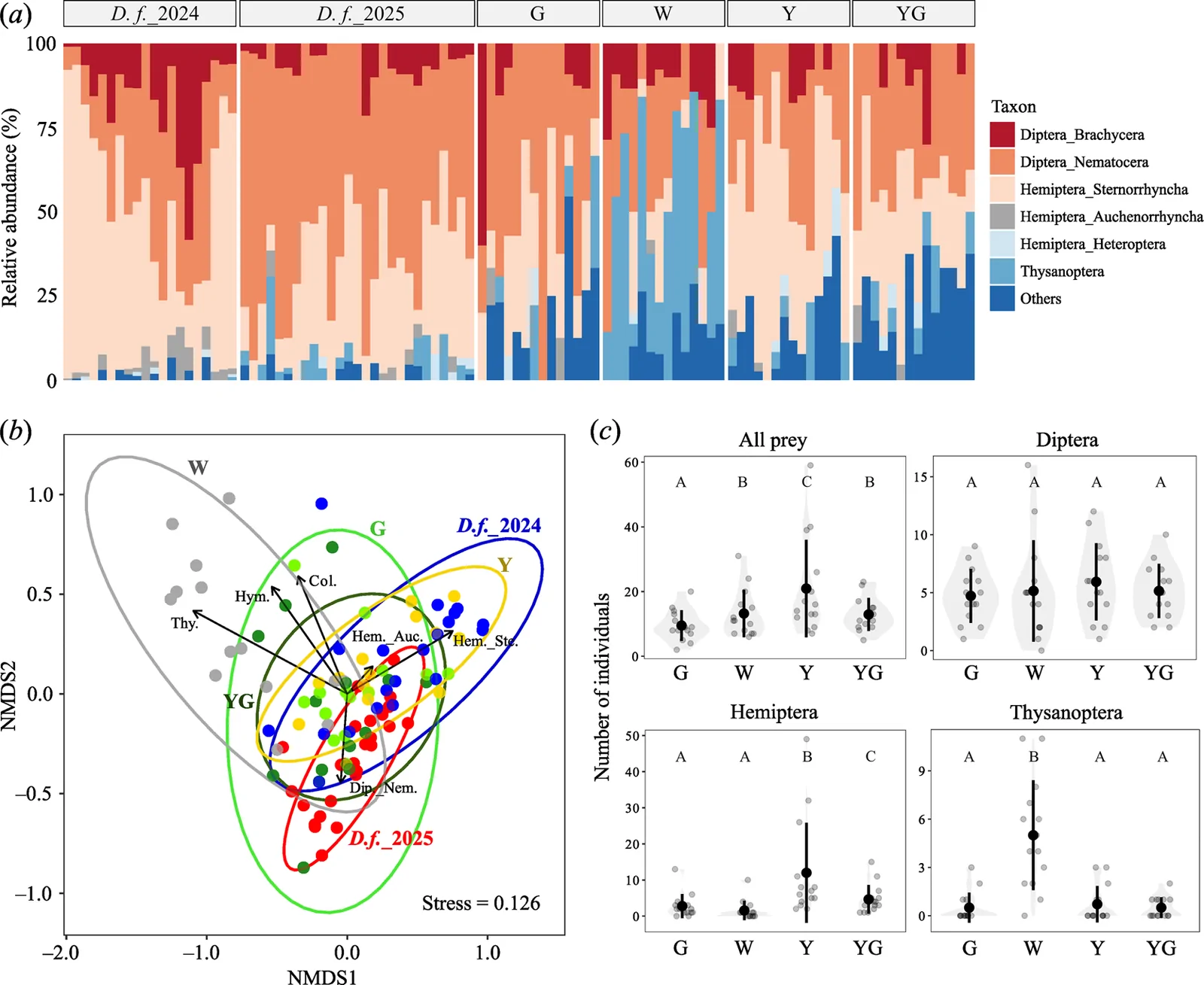
Fauna of arthropods captured by *Drosera finlaysonii* and trap models. (*a*) Taxonomic composition of arthropods captured by individual plants of *D. finlaysonii* and by trap models. G, W, Y and YG indicate trap models coloured green, white, yellow and yellow-green, respectively. Each bar represents one plant or one plant model. Prey taxa other than the major groups (Diptera, Hemiptera and Thysanoptera) are pooled as ‘Others’. (*b*) Non-metric multidimensional scaling (NMDS) ordination based on prey capture data across all prey taxa for *D. finlaysonii* and trap models. (*c*) Violin plots showing comparisons of the numbers of individuals captured by trap models. Light-coloured points represent values for plant models, whereas dark points and lines indicate means ± s.e. Different letters above plots denote statistically significant differences among groups based on generalized linear mixed models with Holm-adjusted multiple comparisons.

**Table 2.** Pairwise Euclidean distances among groups on the NMDS plot for prey captured by *D. finlaysonii* and each plant model (*p*-values from PERMANOVA).

	*D. f.* (2025)	green	white	yellow	mean (all)	mean (model)
*D. f.* (2024)	0.28 (**<0.05**)	—	—	—	—	—
*D. f.* (2025)	—	—	—	—	0.28	—
green	0.14 (**<0.05**)	—	—	—	0.25	0.29
white	0.53 (**<0.05**)	0.48 (**<0.05**)	—	—	0.51	0.50
yellow	0.26 (**<0.05**)	0.24 (**<0.05**)	0.54 (**<0.05**)	—	0.30	0.32
yellow-green	0.20 (**<0.05**)	0.14 (0.34)	0.48 (**<0.05**)	0.17 (0.15)	0.25	0.26

*Note:* Bold text indicates significance levels at *p* < 0.05.

### 3.3. Visual attraction using trap models

The taxonomic composition of prey captured by the trap models differed significantly among colour treatments, except for the comparisons between the green and yellow-green models and between the yellow-green and yellow models (figure 4*a,b*; table 2). Comparisons of mean Euclidean distances between group centroids in the NMDS ordination revealed that the white and yellow models were relatively distant from the other colour models (table 2), indicating that prey assemblages captured by these models were compositionally distinct. Examination of prey composition per plant model (figure 4*a*) and the taxon-specific vectors overlaid on the NMDS ordination (figure 4*b*) showed a strong association between the white model and captures of Thysanoptera, and between the yellow model and captures of Sternorrhyncha (Hemiptera).

For total prey abundance, the yellow model captured significantly more individuals than the other colour models. No significant difference was detected between the white and yellow-green models, whereas the green model captured significantly fewer individuals than all other models (figure 4*c*). For Diptera, no statistically significant differences in capture numbers were detected among colour models, indicating no clear colour bias (figure 4*c*). By contrast, capture numbers of Hemiptera differed significantly among models, with all pairwise comparisons being significant except between the green and white models. Specifically, the yellow model captured the highest number of hemipteran individuals, followed by the yellow-green, green and white models (figure 4*c*). Thysanoptera were captured most frequently by the white model, and capture numbers differed significantly between the white model and all other colour models (figure 4*c*).

Finally, the taxonomic composition of prey captured by *D. finlaysonii* in 2025 differed significantly from that of prey captured by all trap models (figure 4*a,b*; table 2). Thus, the prey-capture pattern of *D. finlaysonii* was not fully reproduced by the yellow model, despite its similar G/B ratio. This difference reflected the lower proportion of Hemiptera and the higher proportion of Diptera in the prey captured by *D. finlaysonii* compared with the prey captured by yellow models (figure 4*a*).

## 4. Discussion

### 4.1. Evidence for EPB in interactions between *D. finlaysonii* and hemipteran prey

EPB has been documented across a wide range of taxa as a mechanism by which predators effectively attract prey. Carnivorous plants may similarly enhance prey capture through EPB, but no previous study has tested this possibility using explicit criteria for EPB (table 1). We evaluated whether the yellowish traps of *D. finlaysonii* enhance the attraction and capture of herbivorous hemipterans through EPB, focusing on the documented preference of these insects for yellow to yellow-green stimuli with high G/B ratios, a perceptual bias arising from the green-blue opponent mechanism and thought to facilitate the location of high-quality food resources (criterion 1).

Our multispectral imaging showed that the trap leaves of *D. finlaysonii* had higher brightness across both visible and UV wavelengths than sympatric graminoid leaves, as well as significantly higher G/B ratios calculated using an aphid visual model (criterion 2). In both 2024 and 2025, *D. finlaysonii* captured hemipterans under natural field conditions (criterion 3), although their proportional contribution declined in 2025. Furthermore, in 2025, the yellow models, which most closely matched the colour of *D. finlaysonii* trap leaves and had the highest G/B ratio, captured more hemipterans than the yellow-green and green models, which more closely matched the colouration of the dominant sympatric graminoids (criterion 4). Taken together, these findings support the hypothesis that the high G/B ratio of *D. finlaysonii* trap leaves contributes to the attraction and capture of hemipterans by exploiting their perceptual bias.

### 4.2. Limits to inferring the contribution of a high G/B ratio to prey capture by *D. finlaysonii*

Nevertheless, the importance of a high G/B ratio in overall prey capture by *D. finlaysonii* remains uncertain. In 2025, the arthropod assemblage captured by *D. finlaysonii* differed significantly from that captured by the yellow models: *D. finlaysonii* captured a lower proportion of Hemiptera and a higher proportion of Diptera. Thus, the similarity in G/B ratio was insufficient to reproduce the observed prey assemblage. Three non-mutually exclusive factors may account for this discrepancy.

First, *D. finlaysonii* may attract and capture dipterans through traits other than the G/B ratio. In both years, *D. finlaysonii* consistently captured large numbers of dipterans, particularly Ceratopogonidae. By contrast, dipteran capture did not differ significantly among model colours, indicating that the colour differences manipulated in this study, variation in brightness and G/B ratio estimated for aphid vision, did not strongly determine dipteran attraction. High capture rates of Ceratopogonidae have been reported repeatedly in other *Drosera* species, including *D. anglica* [57,58] and *D. tracyi* [59]. These species all bear elongated adhesive trap leaves above the ground. This morphology may enhance attraction through strong lateral UV reflectance or may increase capture probability when male ceratopogonids form large mating swarms near wetlands [58]. In addition, many ceratopogonid species are hematophagous or feed on the body fluids of other insects [60], raising the possibility that volatile organic compounds emitted by trap leaves or odours associated with decomposing prey may contribute to attraction [61]. Future studies combining visual and chemical bioassays, as well as field surveys conducted at different times of day and in different seasons, will be necessary to clarify the mechanisms underlying the attraction and capture of Diptera, especially ceratopogonids.

Second, differences in sampling periods may have contributed to the discrepancy between *D. finlaysonii* and the colour models. Arthropods on the models were recorded during the 8-hour deployment period, whereas those on *D. finlaysonii* included all identifiable prey remains present at the time of each survey. The plant data therefore represent a residual assemblage shaped by capture and post-capture loss through decomposition, digestion, rain and wind. Taxon-specific retention times may therefore bias the observed prey composition. In particular, small hemipterans such as whiteflies and aphids may decompose or disappear rapidly and thus have been underestimated.

Third, the prey composition of *D. finlaysonii* may depend on local arthropod availability. Hemiptera predominated in 2024, whereas Diptera predominated in 2025. Moreover, the number of hemipterans captured per plant declined in 2025, whereas no comparable decline was observed for Diptera. Because the visual and trapping traits of *D. finlaysonii* are unlikely to have changed substantially between years, this taxon-specific decline is consistent with a reduction in the local availability of hemipterans rather than a general decrease in plant capture efficiency. However, because local arthropod abundance was not measured directly, this interpretation remains speculative. Thus, even if hemipterans prefer the visual traits of the traps, this preference may not be clearly reflected in prey composition when hemipterans are relatively scarce in the surrounding environment.

### 4.3. Distinguishing model-independent EPB from aggressive mimicry

Although EPB may be a precursor in the gradual evolution of mimicry, further evidence is required to determine whether the present system constitutes aggressive mimicry, with hemipterans as receivers, their food plants as models and trap leaves of *D. finlaysonii* as mimics [1,13]. Perceptual biases arising from structural constraints on sensory systems exist independently of any specific model, and their exploitation alone does not constitute mimicry. Mimicry requires receivers to associate a particular model percept with a specific outcome and mimics to benefit from resembling that percept [12].

Distinguishing these mechanisms would first require identifying candidate food-plant models in the habitat of *D. finlaysonii*. The visual traits of the candidate models, including their G/B ratios, should then be compared with those of *D. finlaysonii* as perceived by the receiver. Experiments using artificial models representing the visual traits of *D. finlaysonii*, candidate food plants and sympatric non-host plants could then test whether the putative mimic and model elicit similar approach and landing responses, and whether these responses change when their perceptual similarity is manipulated.

Responses determined by resemblance to a specific food plant would support aggressive mimicry. By contrast, responses to stimuli with high G/B ratios, regardless of their resemblance to a particular plant, would support model-independent EPB. Although our findings are consistent with model-independent EPB, aggressive mimicry cannot be excluded because model-dependent responses were not tested.

### 4.4. Evolutionary implications of yellow-green traps in carnivorous plants

This study is the first to examine prey attraction in *Drosera* in relation to the visual system of a specific prey group. The anthocyanin-derived red colouration common in the traps of many *Drosera* species has long been hypothesized to attract prey [62,63], but field experiments using red and green models have generally failed to support this hypothesis [64–66]. This is consistent with the limited long-wavelength sensitivity of Diptera [17], which constitute the predominant prey group in many *Drosera* species. By contrast, our results indicate that the high G/B ratio of the bright yellow-green traps of *D. finlaysonii* can contribute to the attraction and capture of hemipterans. Yellowish traps occur in multiple carnivorous plant lineages, including *Nepenthes*, *Sarracenia* and *Pinguicula* [7], and the carnivorous bromeliad *Brocchinia reducta* exhibits strikingly yellow-green leaves compared with its non-carnivorous relative *B. tatei* [67]. Yellow-green colouration may therefore be an overlooked visual trait involved in prey attraction across carnivorous plants.

A high G/B ratio can arise through several physiological mechanisms, each with different costs. One possibility is low chlorophyll content [23]. Carnivorous plants typically inhabit nitrogen-poor environments, where limited nitrogen may constrain chlorophyll production [5,68]. Consequently, yellow-green trap leaves with high G/B ratios may arise as a by-product of nutrient limitation, with their prey-attraction function evolving secondarily. Because carnivorous plants in nutrient-limited habitats are constrained in their ability to invest additional resources in prey attraction, the evolution of specialized attraction signals may also be constrained [14]. However, if insects can be attracted by a high G/B ratio without additional investment in a specialized signal, the evolutionary transition to carnivory may be facilitated. This pathway would, however, involve reduced photosynthetic efficiency [5].

Yellow colouration can also result from increased carotenoid production [69] or reduced anthocyanin content [70]. Carotenoid production requires additional resources [69], whereas reduced anthocyanin content may weaken protection against UV and herbivores [70,71]. Anthocyanin loss may also increase the risk of pollinator capture. Red traps may be inconspicuous to dipteran pollinators with limited long-wavelength sensitivity [65], whereas hoverflies, important pollinators of *Drosera* [72], are attracted to yellow stimuli [19], potentially leading to pollinator–prey conflict [73].

The fact that *D. finlaysonii* has yellow-green rather than fully yellow traps suggests that its visual traits may be optimized through a balance between prey capture benefits and physiological and/or ecological costs. Future work integrating visual traits with measurements of photosynthetic performance, pollinator capture and other fitness components will be essential for a more rigorous understanding of the evolutionary ecology underlying trap-leaf colouration in carnivorous plants.

## Supplementary Material



## Data Availability

Data are available at Figshare [74]. Supplementary material is available online [75].
