# Supplementary material for: Do carnivorous plants exploit prey perceptual bias? A case study in Drosera finlaysonii with yellow-green traps
Source: R Soc Open Sci. 2026 Oct 28;13(10):261740. doi: 10.1098/rsos.261740 (PMC13714396; doi:10.1098/rsos.261740)

Electronic Supplementary Material

table S1. Prey fauna and numbers of individuals captured by trap leaves of *Drosera finlaysonii* and by trap models

Family	Order	*D. finlaysonii*		Trap model, Sep. 2025			
		Sep. 2024 (n = 20)	Sep. 2025 (n = 27)	Green (n = 14)	White (n = 14)	Yellow (n = 14)	Yellow-green (n = 14)
**Araneae**		**1**	**0**	**5**	**3**	**5**	**7**
	Salticidae	0	0	3	3	5	5
	Thomisidae	1	0	0	0	0	1
	NA1	0	0	1	0	0	0
	NA2	0	0	0	0	0	1
	NA3	0	0	1	0	0	0
**Blattaria**		**0**	**0**	**0**	**0**	**0**	**1**
	Blattellidae	0	0	0	0	0	1
**Coleoptera**		**1**	**4**	**1**	**1**	**1**	**3**
	Coccinellidae	1	2	0	1	1	1
	Ptinidae	0	0	0	0	0	1
	Staphylinidae	0	0	1	0	0	0
	NA4	0	2	0	0	0	1
**Collembola**		**0**	**0**	**2**	**3**	**13**	**5**
	NA5	0	0	2	3	13	5
**Diptera (Brachycera)**		**141**	**36**	**10**	**21**	**14**	**11**
	Agromyzidae	0	0	1	1	1	2
	Asilidae	0	0	1	0	0	0
	Calliphoridae	2	0	0	0	0	0
	Dolichopodidae	10	6	1	4	7	4
	Drosophilidae	0	0	1	2	0	0
	Empididae	0	1	0	0	0	0
	Stratiomyidae	77	24	1	10	2	5
	Syrphidae	2	2	3	4	1	0
	NA6	0	1	2	0	3	0
	NA7	44	0	0	0	0	0
	NA8	4	0	0	0	0	0
	NA9	2	0	0	0	0	0
	NA10	0	2	0	0	0	0
**Diptera (Nematocera)**		**278**	**464**	**56**	**48**	**67**	**60**
	Ceratopogonidae	209	428	53	45	62	48

	Chironomidae	34	21	3	2	4	8
	Limoniidae	26	12	0	0	0	0
	Scatopsidae	9	1	0	1	1	4
	Tipulidae	0	2	0	0	0	0
**Hemiptera (Sternorrhyncha)**		**894**	**224**	**35**	**20**	**160**	**60**
	Aleyrodidae	884	221	29	17	145	49
	Aphididae	10	3	6	3	15	11
**Hemiptera (Auchenorrhyncha)**		**31**	**7**	**2**	**1**	**3**	**2**
	Cicadellidae	31	7	2	1	3	2
**Hemiptera (Heteroptera)**		**5**	**6**	**2**	**1**	**5**	**3**
	Lygaeoidea	5	6	0	0	1	1
	Ochteridae	0	0	1	1	2	0
	NA11	0	0	1	0	1	2
	NA12	0	0	0	0	1	0
**Hymenoptera**		**6**	**1**	**6**	**9**	**5**	**9**
	Braconidae	0	0	0	0	1	0
	Eulophidae	0	0	1	0	0	0
	Ichneumonidae	0	0	0	0	0	1
	Mutillidae	0	0	1	0	0	0
	NA13	0	0	0	1	2	2
	NA14	3	0	2	2	0	1
	NA15	1	1	2	6	2	5
	NA16	0	0	0	0	0	1
**Lepidoptera**		**7**	**5**	**0**	**0**	**0**	**0**
	Crambidae	7	5	0	0	0	0
**Orthoptera**		**0**	**1**	**5**	**1**	**5**	**8**
	Acrididae	0	0	1	0	0	1
	Gryllidae	0	0	0	1	0	0
	Pyrgomorphidae	0	0	0	0	1	0
	Rhaphidophoridae	0	1	0	0	0	0
	Tetrigidae	0	0	0	0	1	0
	Tettigoniidae	0	0	4	0	1	2
	Tridactylidae	0	0	0	0	2	5
**Psocoptera**		**0**	**0**	**1**	**4**	**2**	**2**
	NA17	0	0	1	3	2	2
	NA18	0	0	0	1	0	0
**Thysanoptera**		**1**	**25**	**7**	**70**	**10**	**7**
	Thripidae	1	25	7	70	10	7
**Total number**		**1365**	**773**	**132**	**182**	**290**	**178**

figure S1. Yellowish granular structures present on the tentacle stalks of *Drosera finlaysonii*.

(*a*) Trap leaves of *D. finlaysonii*. (*b*) Magnified view of the tentacles (enlarged from the dashed area in panel *a*). Triangles indicate the yellowish granular structures.

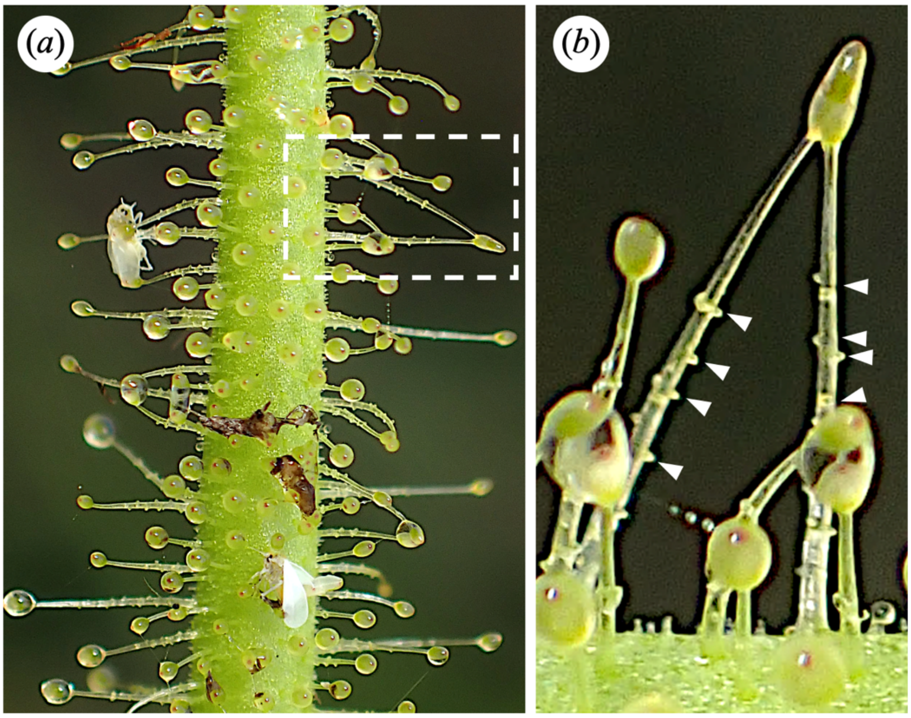

figure S2. Comparison of brightness and G/B ratio among the trap leaves of *Drosera finlaysonii*, leaves of sympatric graminoid species, and trap models of different colors.

The dark points for "*D.f.*" and "Gram." indicate the mean values for the trap leaves of *D. finlaysonii* and leaves of sympatric graminoid species, respectively, whereas the pale points indicate values for individual plants. The points labeled "G", "W", "Y", and "YG" indicate the values for the green, white, yellow, and yellow-green models, respectively.

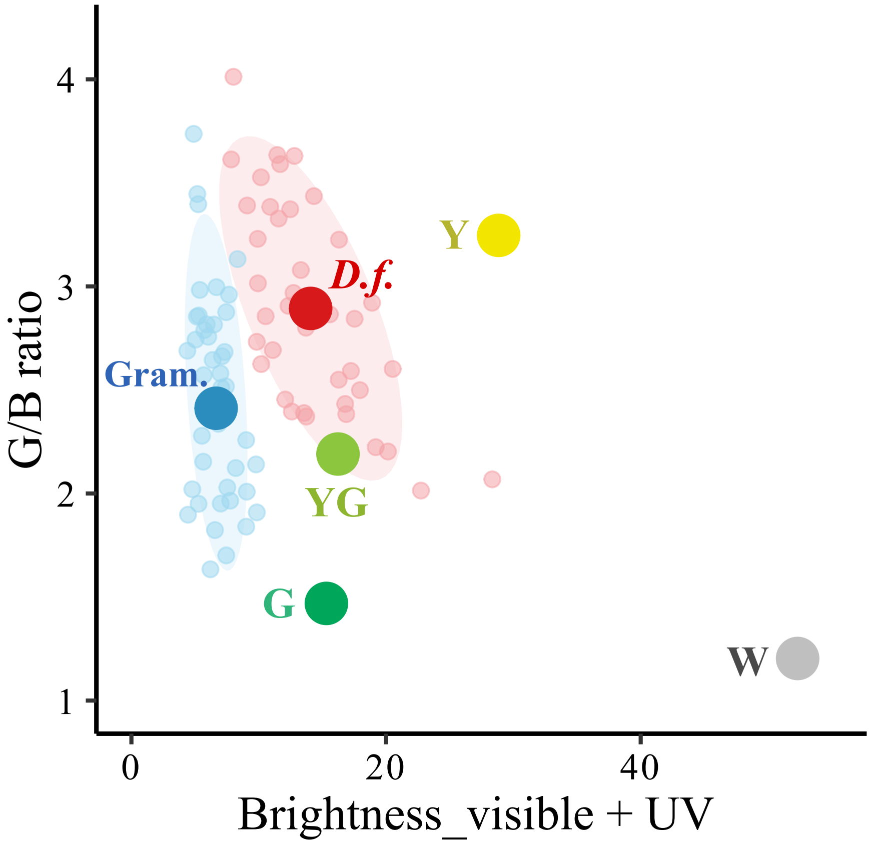

figure S3. Plant models bearing green, white, yellow, or yellow-green trap models deployed at a habitat of *Drosera finlaysonii*.

(*a*) Schematic diagram of the plant model and experimental block. (*b*) Plant models installed next to *D. finlaysonii*.

(*a*)

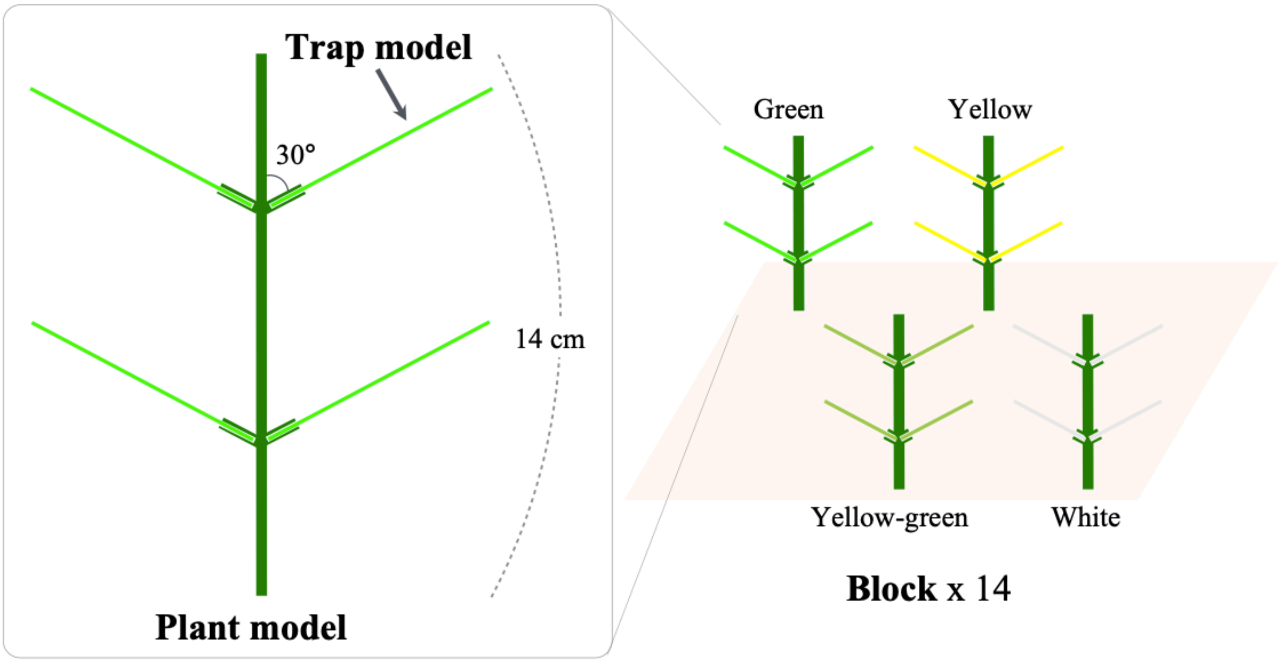

(*b*)

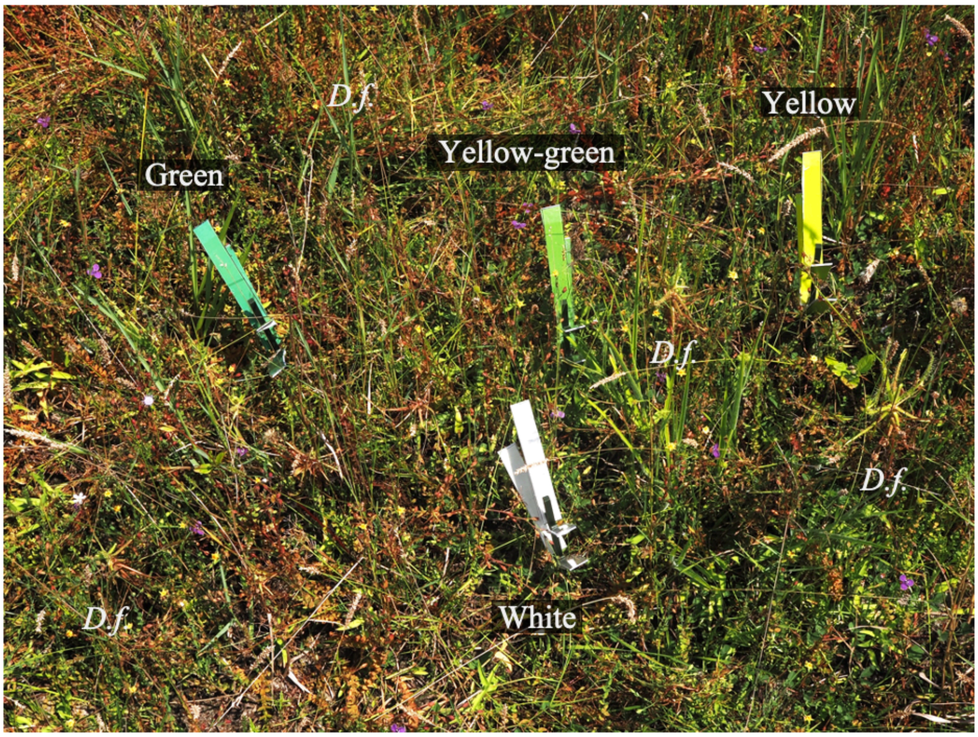

Supplement: Supplementary file 1 [file rsos.261740.zip › rsos.261740/rsos261740_si_001.pdf]
